# Measurements of hydrocortisone and cortisone for longitudinal profiling of equine plasma by liquid chromatography–tandem mass spectrometry

**DOI:** 10.1002/dta.3244

**Published:** 2022-03-04

**Authors:** Kathy Tou, Adam Cawley, Christopher Bowen, Kireesan Sornalingam, Shanlin Fu

**Affiliations:** ^1^ Centre for Forensic Science University of Technology Sydney Broadway New South Wales Australia; ^2^ Australian Racing Forensic Laboratory Racing NSW Sydney New South Wales Australia; ^3^ Mass Spectrometry Business Unit Shimadzu Scientific Instruments (Australasia) Rydalmere New South Wales Australia

**Keywords:** biomarker, cortisone, equine plasma, hydrocortisone, longitudinal profiling

## Abstract

The conventional detection of exogenous drugs in equine doping samples has been used for confirmation and subsequent prosecution of participants responsible. In recent years, alternative methods using indirect detection have been investigated due to the expanding number of pharmaceutical agents available with the potential of misuse. The monitoring of endogenous biomarkers such as hydrocortisone (HC) has been studied in equine urine with an international threshold of 1 μg/ml established; however, there is no current threshold for equine plasma. The aim of this research was to investigate plasma concentrations of HC and cortisone (C) in race day samples compared to an administration of Triamcinolone Acetonide (TACA). The reference population (*n* = 1150) provided HC (6 to 145 ng/ml) and C (0.7 to 13 ng/ml) levels to derive the HC to C ratio (HC/C). Population reference limits (PRLs) were proposed for HC/C values at 0.2 (lower) and 61 (upper). Administration of TACA resulted in down‐regulation of HC/C values below the estimated PRLs for up to 96 h post‐administration. This indirect detection period was longer than the detection of TACA for 72 h. The use of individual reference limits (IRLs) for HC/C values was investigated to support the Equine Biological Passport (EBP), an intelligence model developed by Racing NSW for longitudinal monitoring of biomarkers.

## Graphical abstract

Alternative methods of substance uses are being investigated to complement conventional target‐based screening. The monitoring of hydrocortisone and cortisone was investigated in equine plasma as there is currently no threshold. Plasma hydrocortisone and cortisone levels in race day samples were compared to those observed following administration of Triamcinolone Acetonide. The use of individual reference limits was also applied to examine the potential for longitudinal monitoring of the plasma hydrocortisone to cortisone ratio.

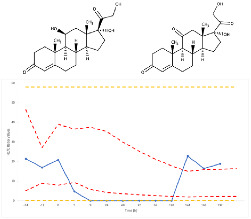

## 1 INTRODUCTION

The use of performance enhancing drugs is a major issue for equine sports. With the increasing number of new substances being rapidly developed, the need for complimentary methods for detection and monitoring is important to ensure the integrity of sport.^1^, ^2^ The use of biomarkers for the detection of doping abuse is a novel yet significant advancement for sports anti‐doping.^3^ Teale et al. defined biomarkers as an ‘individual biological parameter or substance (metabolite, protein or transcript); the concentration of which is indicative of the use or abuse of a drug or therapy’.^3^ Biomarker concentrations are reliant on an individual's physiological parameters, and responses to stimuli.

The concept of an athlete biological passport (ABP) was introduced by the World Anti‐Doping Agency (WADA) for haematological monitoring^4^, ^5^ in 2009 and expanded with the Steroidal Module in 2014.^6^, ^7^ For equine sports, Duluard et al. investigated longitudinal profiling for French trotting racehorses over 1 year to establish individual profiles for interpretation of data that may be suspicious during the racing season.^8^ This model was applied by Racing NSW to develop the Equine Biological Passport (EBP). The EBP consists of a number of horses subjected to longitudinal monitoring of biomarkers, which allows for the detection of performance enhancing substances by measuring changes.^9^ The EBP is considered part of a forensic intelligence model as it enables for the collection of analytical information together with veterinary advice, betting market analysis and stewards reviews.^9^ This can be used by authorities to monitor specific horses who display suspicious profiles.^2^, ^9^

Historically, in the racing industry thresholds are established using population reference limits (PRLs) derived from clinical chemistry applications.^10^, ^11^ However, as the endogenous levels of biomarkers are known to vary between individuals, PRLs can be insensitive to sub‐threshold levels that have originated from doping. Utilising a parametric empirical bayes (PEB) algorithm reported by McIntosh et al. for cancer research, individual reference limits (IRLs) can more effectively monitor up‐ or down‐regulation of biomarkers.^12^, ^13^

Hydrocortisone (HC; also commonly known as cortisol) is a primary glucocorticosteroid that is naturally secreted by the adrenal cortex.^14^, ^15^ It is used by the body to limit the response to stress, reduce inflammation and increase the availability of glucose to induce the feeling of euphoria.^16^ In horses, HC can be used artificially as a replacement therapy for adrenal deficiency and to treat inflammation in joints of horses.^14^ The current threshold for HC is only established for urine at 1 μg/ml.^17^, ^18^ There are limitations for the interpretation of HC levels in racehorses due to circadian rhythm observed between morning (peak concentration) and night (lowest concentration).^19^ Held et al. observed that following dexamethasone administration circadian rhythm can influence the return to basal HC levels.^19^ Such variation may have contributed to the lack of a current HC threshold in plasma for equine sports.

Triamcinolone Acetonide (TACA) is a synthetic corticosteroid that is approximately 5 times more potent than HC as an anti‐inflammatory agent^20^ and as an effective analgesic.^21^ It is commonly used for the management of exercise‐associated articular osteoarthritis as it is less detrimental to the health of joints in the horse.^20^ The current rules controlling the use of intra‐articularly administered corticosteroids in Australia (AR 87) states that treatment must not be performed on horses at least 8 days prior to racing.^22^ The effects of TACA are known to suppress various endogenous compounds including HC.^20^ HC suppression has also been observed following administration of other synthetic corticosteroids, such as flumetasone and dexamethasone.^23^, ^24^, ^25^

The aim of this study was to investigate upper and lower thresholds for HC levels in plasma, and the potential use of cortisone (C) as an endogenous reference compound (ERC) to account for physiological variation. C is an endogenous corticosteroid that displays anti‐inflammatory properties.^26^ Results from a reference population of routine pre‐race doping control samples were compared to TACA administrations. The potential for intra‐individual (longitudinal) profiling of HC and HC/C values to improve the detection of abnormalities as part of an intelligence‐based anti‐doping strategy was also investigated.

## 2 METHODS

### 2.1 Chemicals and reagents

Acetonitrile (ACN), dichloromethane (DCM), di‐isopropyl ether (DIPE), ethanol (EtOH), ethyl acetate, formic acid, hexane and methanol (MeOH) were purchased from Merck (Darmstadt, Germany). Trichloroacetic acid (TCA) was obtained from Sigma Aldrich (Castle Hill, NSW, Australia). Water used was ultrapure grade from a Thermo Scientific Barnstead Smart2Pure water purification System (ThermoScientific; Langenselbold, Hungary). All solvents used were of HPLC grade.

Certified reference standards for HC and C were obtained from Sigma Aldrich (Castle Hill, NSW, Australia). Internal standard D_4_‐HC was obtained from Cambridge Isotope Laboratories (Andover, Massachusetts, USA). TACA was obtained from Toronto Research Chemicals (Toronto, Ontario, Canada).

### 2.2 Routine race day blood samples

The use of race day samples for reference population profiling was approved by the Racing NSW Animal Care and Ethics Committee (ARA 71). Blood samples (*n* = 1150) from thoroughbred horses were collected prior to race by veterinarians employed by Racing NSW using Lithium Heparin 10‐ml Vacutainer tubes (Becton‐Dickinson, North Ryde, NSW, Australia). Samples were refrigerated and transported to the Australian Racing Forensic Laboratory (ARFL) where, on arrival they were centrifuged at 3000 × g to ensure separation of the plasma layer and stored at 4°C in their respective Lithium Heparin tubes until an aliquot was taken for analysis.

### 2.3 Triamcinolone Acetonide administration

Animal ethics approval was obtained from the Charles Sturt University (Wagga Wagga, NSW, Australia) Ethics Committee. Three thoroughbred geldings (ages of 5 to 8, weight 450 to 550 kg) were each administered 2.0 ml of Kenocort‐A® (Bristol‐Myers Squibb; TACA [20 mg] as an aqueous mixture) via intra‐articular (IA) injection into the left fetlock joint. Blood samples were collected in 6‐ml Lithium Heparin Vacutainer tubes 24 h prior to administration followed by 0 (time of administration), 5‐, 9‐ and 24‐h post‐administration then daily for 8 days. Plasma was obtained by centrifugation (3000 × g) immediately after collection and stored at −20 °C until analysis.

### 2.4 Preparation of standards

Intermediate and working solutions of HC and C were prepared at 20 and 2 μg/ml in MeOH. The internal standard, D_4_‐HC was also prepared at 2 μg/ml in MeOH.

### 2.5 Preparation of surrogate matrix

Pooled blank plasma was obtained from blood samples collected in 10‐ml Lithium Heparin Vacutainers from four research gelding horses following approval from the Racing NSW Animal Care and Ethics Committee (ARA 71). The pooled plasma was screened using routine methods at the ARFL to determine that the plasma did not contain prohibit substances. Using the previous work of Popot et al. investigating urinary equine HC levels, the surrogate matrix was prepared following liquid–liquid extraction of 3‐ml aliquots with DCM/EtOH (90:10 v/v, 4 ml).^27^ The combined aqueous layers were stored at 4 °C for up to 2 months.

### 2.6 Sample preparation

Plasma samples (2 ml) were fortified with D_4_‐HC (25 ng/ml) before the addition of TCA (10% v/v, 200 μl). Samples were diluted with water and pH was adjusted to between 3 and 3.5 using dilute HCl (3% v/v). Samples were centrifuged at 3000 × g for 10 min to separate the protein pellet and obtain the supernatant for solid phase extraction (SPE).

SPE was completed using a positive pressure manifold and XTRACKT (200 mg, 3 ml) Gravity Flow DAU cartridges (UCT; Bristol, PA, USA) in accordance with the routine ARFL method for analysis of equine plasma samples. The cartridges were conditioned with MeOH and water. Samples were loaded, washed with acetic acid, and purged with nitrogen. The acidic‐neutral fraction was eluted using a solution of ethyl acetate/hexane (3:2 v/v, 3 ml). Each sample was dried under nitrogen and reconstituted in 0.1% formic acid/methanol (50 μl) and 0.1% formic acid/water (50 μl). Samples were then transferred to liquid chromatography (LC) vials and stored at 4 °C until analysis.

### 2.7 Liquid chromatography‐mass spectrometry analysis

Liquid chromatography–tandem mass spectrometry (LC–MS/MS) analysis was undertaken using a Nexera X2 UHPLC coupled to an 8060 triple quadrupole mass spectrometer (Shimadzu, Kyoto, Japan). The LC system was equipped with a Shimpack C18 ODS III (75 mm × 2.0 mm, 10 μm) column maintained at 60°C. Mobile phase A consisted of aqueous ammonium formate (5 mM, pH 3.0) and mobile phase B consisted of 0.1% formic acid in ACN. The run time for each sample was 17.5 min with the gradient: 0–2.5 min (A: B 70:30 v/v), 2.5–11 min (A: B 40:60 v/v), 11–12 min (A: B 5:95 v/v) and this was kept constant until 16 min. The mobile phase returned to initial condition for 1.5 min. The flow rate was 0.3 ml/min and an injection volume of 5 μl was used.

Mass spectrometry (MS) data for HC, C and D_4_‐HC were acquired in positive electrospray ionisation (ESI+) mode while the quantification of TACA as formate adduct was performed using negative electrospray ionisation (ESI‐) mode, following routine procedures at ARFL. Collision energies were optimised for each of the compounds. The desolvation line and heat block temperature was set to 250 °C and 400 °C, respectively. The nebulising gas flow, heating gas flow and drying gas flow was set to 2.8, 12.0 and 8.0 L/min respectively.

### 2.8 HC, C and TACA quantification

The calibration range and QCs were prepared using a surrogate matrix as explained in Section 2.5.^27^ Calibration was performed based on previous work by Soma et al.^20^; 0 to 200 ng/ml for HC and 0 to 10 ng/ml for C. QC levels were 10, 50 and 100 ng/ml for HC, and 1, 5, and 10 ng/ml for C (refer to Tables S1 and S2). Sensitivity, linearity, accuracy, precision, recovery, matrix effects and stability were assessed for the three compounds studied. Dilution was assessed for HC only. Details of these are provided in Section S1. Multiple reaction monitoring (MRM) was performed according to Table 1.

**TABLE 1:** MRM acquisition parameters for the three targeted analytes and the two internal standards

Compound (ESI polarity)	Precursor ion (*m/z*)	Product ions (*m/z*)	Collision energy (eV)
**Hydrocortisone (+)**	363.1	121.2	−21
		327.8	−17
		145.2	−33
**Cortisone (+)**	361.2	163.1	−23
		121.1	−30
**D**_**4**_ **‐Hydrocortisone (+)**	367.2	121.2	−26
**TACA (Formate adduct [‐HCOO])**	479.5	413.3	20
		375.3	18
		337.2	25
**D**_**7**_ **‐TACA (‐HCOO)**	486.5	420.2	24

### 2.9 Data processing

#### 2.9.1 LC–MS/MS

Data was analysed using Shimadzu LabSolutions Insight application (version 3.2). Concentrations of HC, C and TACA were determined using linear regression.

#### 2.9.2 Reference population

Statistical functions were performed using Microsoft Excel and MATLAB (Version R2021a). Parametric distributions were evaluated using the Anderson‐Darling test after log transformation for application of proposed population reference limits (PRLs) (Equation 1) at the 99.99% confidence level, representing a natural exceedance of 1 in 10,000.

(1) $$\mathrm{PRL}=\text{Mean}\pm \left(\text{Standard Deviation}\times 3.72\right)\;$$

#### 2.9.3 Individual reference limits

The application of IRLs for HC/C values was investigated for the EBP. Upper and lower IRLs were estimated using Equations (2), (3), (4).^12^, ^13^

(2) $$\text{Upper Reference Limit}=\mu +\left(\bar{x}-\mu \right)B_{n}+z_{\alpha }\sqrt{1-B_{1}B_{n}}\;\sqrt{V}$$

(3) $$\text{Lower Reference Limit}=\mu +\left(\bar{x}-\mu \right)B_{n}-z_{\alpha }\sqrt{1-B_{1}B_{n}}\;\sqrt{V}\;$$

(4) $$\mathrm{Bn}=\frac{\sigma^{2}}{s^{2}/n+\sigma^{2}}$$

Briefly, these equations can be described by μ (population mean), $\bar{x}$ (individual mean), *B*_*n*_ is the intra‐ class correlator, z_α_ is the confidence interval applied and *V* is the total variance. As *n* increases, the proportion of individual variance increases, and population variance decreases. The coverage interval used for the IRLs is 3.09, which represents a confidence level of 99.9% (i.e., natural exceedance of 1 in 1000).

## 3 RESULTS AND DISCUSSION

### 3.1 Method validation

#### 3.1.1 Quantification of HC and C

Due to the endogenous content of both HC and C in plasma, these compounds were removed to produce a surrogate matrix suitable for preparation of calibration and QC samples (Section 2.5). For the produced surrogate matrix, 99.99% of HC and 100% of C was removed (Table S4). This enabled correlation coefficients of 0.99 or greater, without observed bias evaluated from residual analysis. The LOD (estimated by *S/N* > 3) was < 0.05 ng/ml for HC and 0.05 ng/ml for C. The LOQ (estimated by *S/N* > 10) was < 0.05 ng/ml for HC and 0.10 ng/ml for C. Due to the relatively high endogenous content of HC, the estimated LOD and LOQ are less precise.

The accuracy, precision, recovery and matrix effects for HC and C measurements were assessed at three QC concentrations in seven replicates (Table 2 for HC and C). Dilution was assessed for HC (Table 3). HC and C were deemed to be stable for 3 months at both 4 °C and −20 °C.

**TABLE 2:** Method validation results for HC and C

	Quality control	Accuracy (% RE) (*n* = 30)	Precision (% RSD) (*n* = 30)	Recovery (%) (*n* = 7)	Matrix effects (%) (*n* = 7)
**Hydrocortisone**	LQC (10 ng/ml)	−32	24	74	46
	MQC (50 ng/ml)	−11	6.7	58	70
	HQC (100 ng/ml)	−8.9	6.9	54	79
**Cortisone**	LQC (1 ng/ml)	−17	15	51	71
	MQC (5 ng/ml)	5.2	8.0	50	67
	HQC (10 ng/ml)	10.6	7.4	50	72

**TABLE 3:** Dilution assessment for HC only

Dilution	Average dilution concentration (ng/ml)	Actual concentration (ng/ml)	% RE
**1 in 2 (1 ml)**	133	266	11
**1 in 4 (0.5 ml)**	66	265	12

For HC values, both MQC and HQC were within acceptable limits for accuracy (11% and 8.9%, respectively) and precision (6.7% and 6.9%, respectively). Recovery was estimated at 58% and 54% and matrix effects were evident with ion suppression. For HC LQC, accuracy and precision were estimated at 32% and 24% respectively, most likely due to the endogenous content. Recovery was estimated at 74% with evidence of matrix effects due to ion suppression. For C, recovery was estimated at 50% and 51% while matrix effects were also evident due to ion suppression at all QC levels. Accuracy and precision were within 20%.

#### 3.1.2 TACA

Calibration of TACA was performed using pooled plasma to provide a correlation coefficient of greater than 0.99 and residual analysis showing no observed bias. The LOD (estimated by *S/N* > 3) was 0.02 ng/ml and the LOQ (estimated by *S/N* > 10) was 0.05 ng/ml. TACA was deemed to be stable after 3 months at both 4 °C and −20 °C. Accuracy, precision, recovery, and matrix effects for TACA measurements were assessed at QC concentrations 0.05 and 1 ng/ml from seven replicates (Table 4).

**TABLE 4:** Method validation results for TACA

	Quality control (ng/ml)	Accuracy (% RE)	Precision (% RSD)	Recovery (%)	Matrix effects (%)
**TACA**	0.05	−41	30	60	110
	1	−5.6	7.9	51	78

### 3.2 Reference population

**FIGURE 1:**
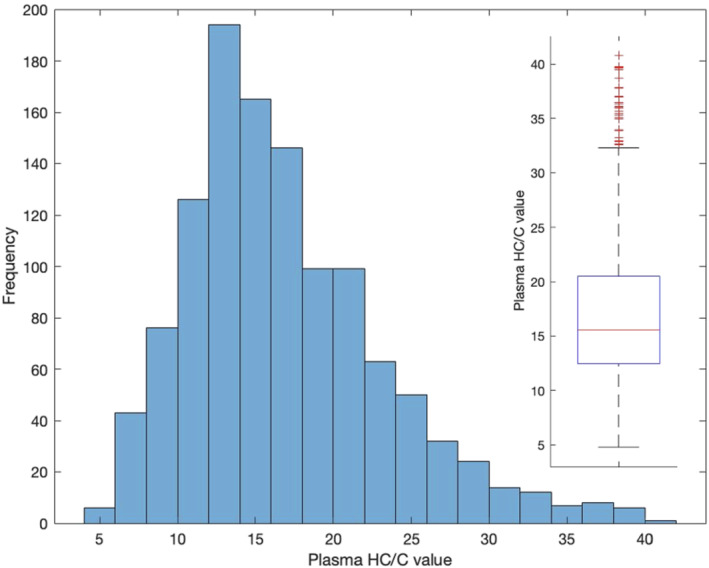
Frequency distribution for untransformed HC/C values in equine plasma (inset: Boxplot providing nonparametric range and estimation of outliers) (*n* = 1150)

A total number of 1150 race day samples were assessed for basal concentrations of HC (Table S5) and C (Table S6) to determine HC/C values. C measurements were quantifiable in the range of 0.8 to 10 ng/ml. Therefore, the use of C as an ERC was investigated to produce a ratio value between HC and C to normalise results for physiological and analytical variation. Figure 1 shows the frequency distribution for HC/C values in equine plasma. The mean was 16.9, median was 15.6 and range was between 4.80 to 40.8. Outliers were estimated using the first and third quartile of 12.5 and 20.5. Refer to Section S2, Figures S1 and S2 for HC results and Figures S3 and S4 for C results.

**FIGURE 2:**
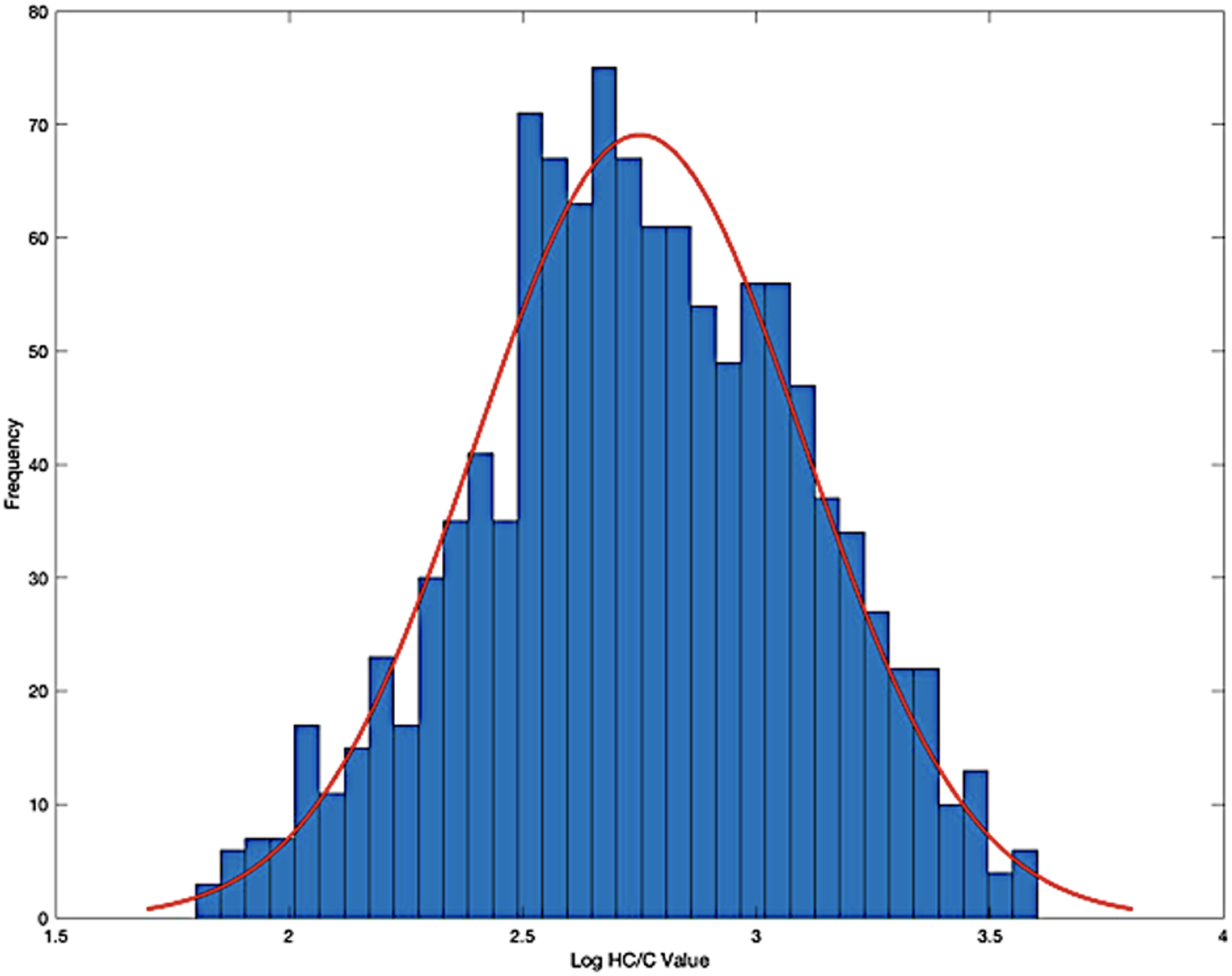
Log normal transformed frequency distribution for HC/C values in equine plasma (*n* = 1150)

Since the untransformed values for both HC and HC/C did not display a parametric distribution, a logarithmic transformation was performed. Figure 2 shows the fitted lognormal distribution for HC/C values.

Plasma HC values displayed a lognormal distribution for 81% of all samples while HC/C values had a parametric range covering 98% of the sample set (Figure S5). A parametric distribution for log HC/C values was further supported by the Anderson–Darling test (*p* = 0.05). This demonstrated the potential advantage of applying HC/C values to propose PRLs and IRLs.

Results from the reference population were reviewed between genders to show a potential difference (*p <* 0.05). This is illustrated in comparative boxplots provided in Figures S6 and S7. For Figure S6, a comparison was completed using the number of samples obtained originally between geldings (*n* = 475), females (*n* = 447) and males (*n* = 228). To reduce the potential effect of sample size, a comparison between the three genders with 228 samples randomly selected for geldings and females was performed to provide a likely difference (*p* < 0.05) between gender (Figure S7).

Notwithstanding this, the log transformed HC/C values were used to propose PRLs according to Equation 1. Summary statistics are provided in Table 5.

**TABLE 5:** Summary statistics for HC/C values in equine plasma (*n* = 1150)

	Gelding	Female	Male	Combined
**Total number of samples for each gender**	475	447	228	N/A
**Mean**	16.4	15.7	18.9	16.7
**Standard Deviation**	5.55	5.59	6.35	5.85
**Range**	6.70 – 35.3	6.13 – 35.7	7.22 – 35.9	6.13 – 35.9
**Log Transformed Upper Value**	3.99	4.02	4.14	4.06
**Log Transformed Lower Value**	−1.49	−1.36	−1.62	−1.44
**Proposed Upper PRL**	54.3	55.8	63.0	57.9
**Proposed Lower PRL**	0.22	0.26	0.20	0.24

### 3.3 Longitudinal profiling for EBP

**FIGURE 3:**
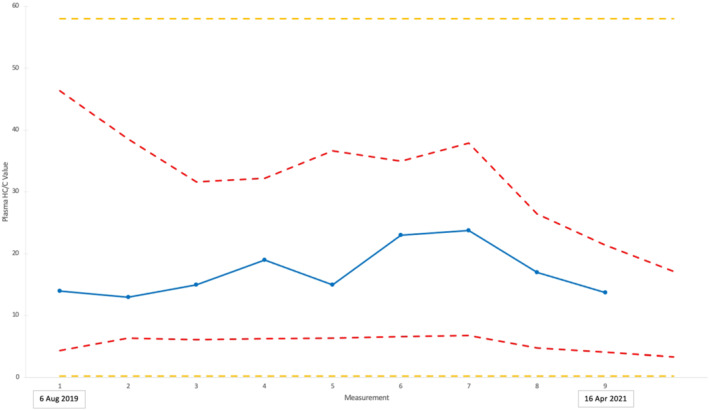
Equine Biological Passport (EBP) profile for Horse 1. Plasma HC/C values (blue line), upper and lower IRLs (red dotted lines) and proposed upper and lower PRLs (yellow dotted lines)

**FIGURE 4:**
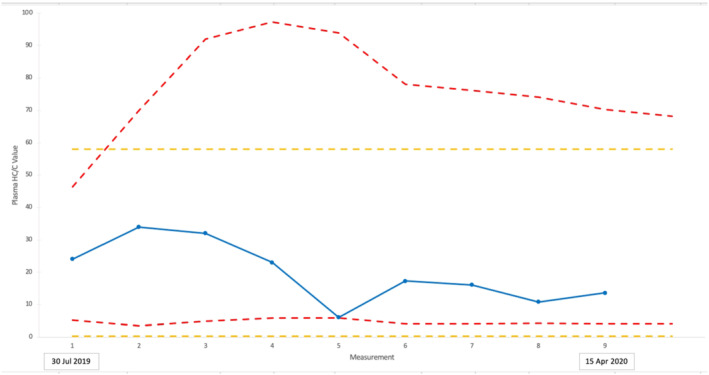
Equine Biological Passport (EBP) profile for Horse 2. Plasma HC/C values (blue line), upper and lower IRLs (red dotted lines) and proposed upper and lower PRLs (yellow dotted lines)

Two horses were used to evaluate the potential for HC/C values to be included in longitudinal profiling as part of the EBP. Applying Equations (2), (3), (4) results in IRLs that commence at *n* = 1 with population‐derived values, representing the 99.9% confidence level. Figures 3 and 4 show how this compares with the 99.99% confidence level used to propose the PRL.

Figure 3 shows Horse 1 to display relatively stable HC/C values that did not challenge the estimated IRLs. This horse was monitored as part of the EBP from August 2019 to April 2021 with a total of nine samples.

Horse 2 in comparison displayed (Figure 4) greater variance in HC/C values, including an abnormal value for sample 5, equivalent to the lower IRL of 6.0, collected on 20 February 2020. In comparison to the PRL, sample 5 would not have been identified as abnormal. In addition to the forensic intelligence an IRL provides when the samples are consistent, there is also a form of intelligence when the IRL exceeds the PRL. After the first sample from Horse 2, the IRL is higher than the PRL due to the standard deviation between the samples being greater than 0.4. In this case, the estimated upper IRL is abnormal (being greater than the PRL). This may indicate evidence of doping or an unknown health issue in the horse displayed early in the profile that requires further investigation by stewards and veterinarians. Nevertheless, the potential of biomarker assessments using IRLs as part of an intelligence‐based anti‐doping strategy is illustrated.

### 3.4 TACA administration

**FIGURE 5:**
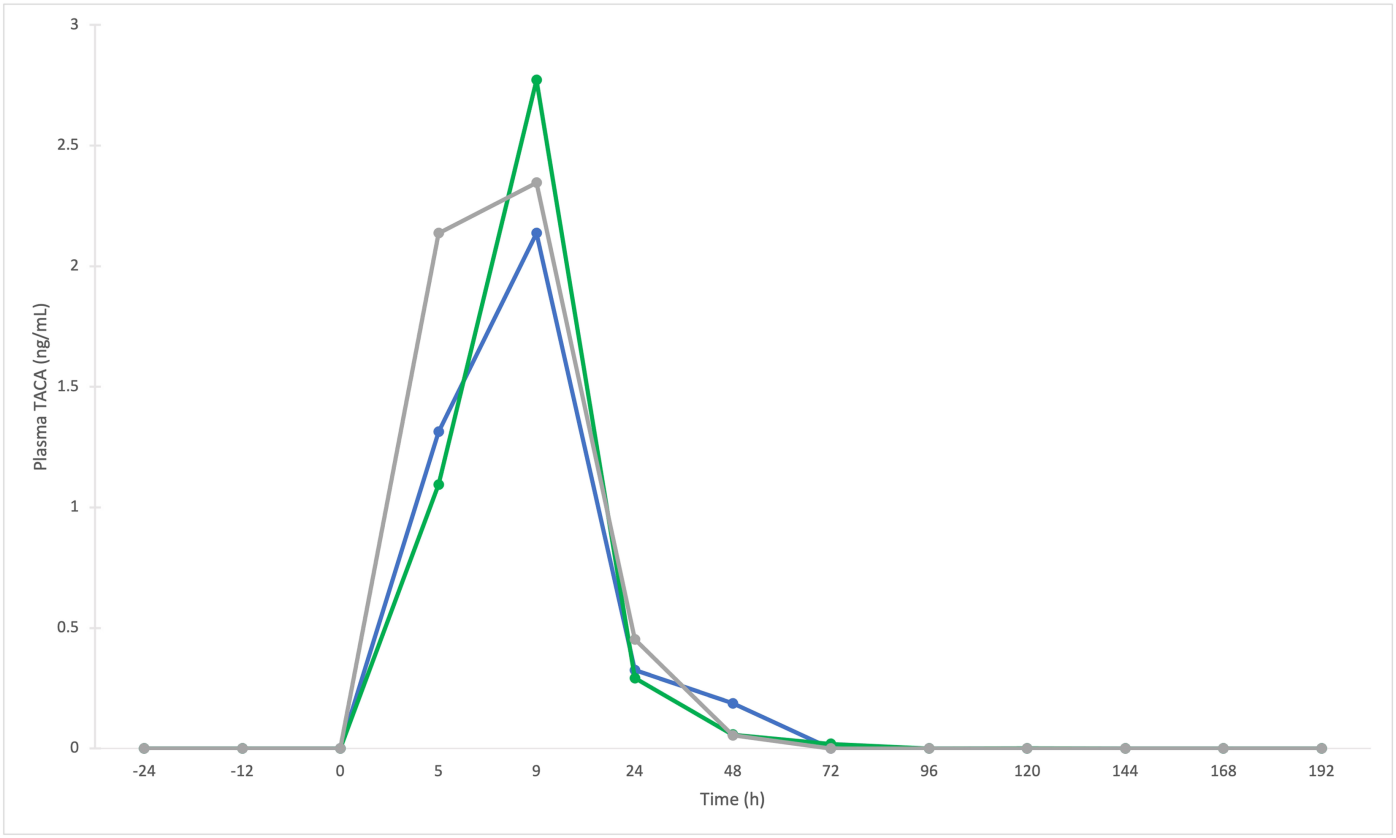
Plasma elimination profile of Triamcinolone Acetonide (TACA) (20 mg IA) for Horse 3 (blue line), 4 (green line) and 5 (grey line)

**FIGURE 6:**
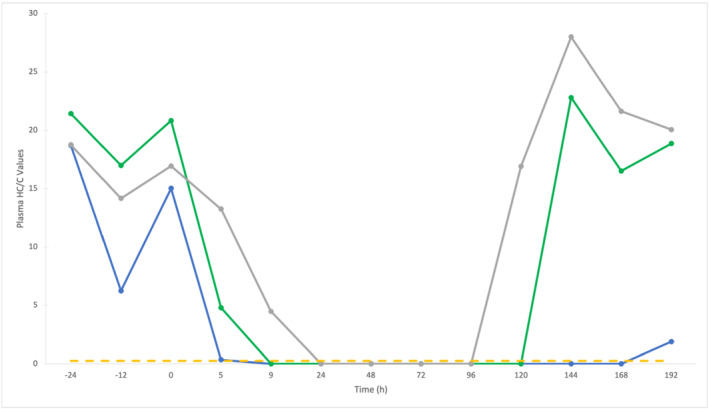
Plasma HC/C values before and after administration of Triamcinolone Acetonide (TACA) Horse 3 (blue line), 4 (green line) and 5 (grey line). Yellow dotted line represents the proposed lower population reference limit (PRL) (0.2)

TACA administration studies were analysed to investigate the effect on plasma HC/C values. Plasma profiles of TACA, HC and HC/C for Horses 3, 4 and 5 are shown in Figures 5 and 6. Refer to Figure S8 for HC concentrations following TACA administration.

TACA was detected in all three horses between 5 and 72 h post‐administration. The maximum TACA concentrations were determined to be 2.1 ng/ml (Horse 3), 2.8 ng/ml (Horse 4) and 2.3 ng/ml (Horse 5) at 9 h post‐administration. HC/C values varied during TACA elimination. For Horse 3, the basal HC/C was approximately 18 with down‐regulation observed at 9 h post‐administration, below the PRL of 0.24, which remained until 168 h post‐administration. For Horse 4 and Horse 5, basal HC/C values were calculated to be at 21 and 19 respectively, with down‐regulation beyond the PRL observed until 120 h post administration for Horse 4 and between 24 and 96 h post‐administration for Horse 5. At 144 h post‐administration, Horse 5 displayed evidence of positive feedback to achieve homeostasis with a HC/C value of 28, before returning to 20 at the final time point of 192 h. Using HC/C results, the detection period for TACA administration increased from 3 days to between 4 and 8 days, all be it indirectly.

The utility of this approach may be explored for other routes of TACA administration. Soma et al. investigated the effects of endogenous plasma HC under the influence of TACA^20^ and compared intramuscular (IM), intravenous (IV) and IA administrations. The route of TACA administration displayed no significant influence on plasma HC concentrations or the time that minima were observed, however it did affect the time required for return to baseline.^20^

Furthermore, the broad application of HC/C profiling is promising to increase the detection period for other corticosteroids. Knych et al. explored the effects of flumetasone IV on plasma HC concentrations to show down‐regulation for up to 72 h post‐administration compared to the direct detection period of 36 h.^23^ This was also the case for dexamethasone with reduced HC levels observed to be longer than the detection of dexamethasone itself.^24^ Soma et al. continued work on dexamethasone that further supported the use of plasma biomarkers to determine the duration of action for an exogenously administered corticosteroid in the horse.^25^

**FIGURE 7:**
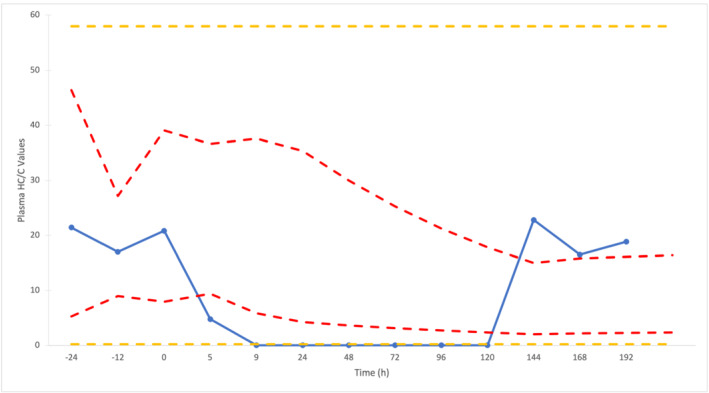
Profile of plasma HC/C values before and after administration of Triamcinolone Acetonide (TACA) for Horse 4 with comparison to individual reference limits (IRLs) (red dotted lines) and population reference limits (PRLs) (yellow dotted lines)

The investigation of HC/C values following corticosteroid administration of TACA also provided the opportunity to assess the intelligence value of IRLs compared to PRLs. Horse 4 was used as an example to illustrate this (Figure 7) due to the profile showing both up‐ and down‐regulation. The profiles for Horses 3 and 5 are provided in Figures S9 and S10, respectively. The three measurements obtained prior to TACA administration provided a reasonable profile for basal levels in relation to the upper PRL for HC/C of 58 and lower PRL of 0.2. The latter is breached between 9 and 120 h. Using IRLs, there is evidence of abnormalities based on both down‐ and up‐regulation of HC/C values. The “intelligence” period between 5 to 144 h (i.e., 139 h) post‐administration is 72 h longer than the direct detection period (5 to 72 h) for TACA.

There are limitations for implementing EBP profiles using IRLs. The primary disadvantage could be the potentially short duration of a race horse's career in comparison to human athletes.^9^ Notwithstanding this, the EBP can serve as a deterrent for the use of prohibited substances since they may leave a biological signature that exists beyond the detection of the substance itself.^9^

## 4 CONCLUSION

Current direct methods of detection for exogenous drugs have been effective over the past 40 years. In the field of equine anti‐doping however, there is an ever‐growing list of exogenous drugs available, which requires alternate methods of detection to monitor endogenous biomarkers. As the current international threshold stands at 1 μg/ml for HC in urine, there is a need to better understand HC measurements in equine plasma. However, the influence of circadian rhythm may limit the use of HC levels alone. Analysis of routine race day samples (*n* = 1150) revealed a mean HC concentration of 56 ng/ml and a range between 0 and 10 ng/ml for C. The use of C as an ERC to derive HC/C values is proposed to better account for individual variation and provide greater retrospective evidence of corticosteroid administration. Following TACA administration, the HC/C ratio indirectly extended the detection period from 72 to 192 h. Furthermore, the use of IRLs from longitudinal profiling was shown to be advantageous for intelligence purposes in addition to the conventional consideration of PRLs. There is potential for broader applications of IRLs estimated for HC/C to monitor other corticosteroid administrations, including those that may not be specifically targeted.

## Supporting information




**Table S1:** Plasma spikes for calibration of Hydrocortisone (HC)
**Table S2:** Plasma Spikes for Calibration of Cortisone (C)
**Table S3:** Plasma Spikes for Calibration of Triamcinolone Acetonide (TACA)
**Table S4:** Removal of endogenous compounds using DCM: EtOH (90:10)
**Table S5:** Reference Population Data for HC:
**Table S6:** Reference Population Data for C:
**Figure S1:** Frequency Histogram with fitted gaussian distribution for Hydrocortisone values
**Figure S2:** Log normal transformed frequency histogram with fitted gaussian distribution for hydrocortisone values
**Figure S3:** Frequency histogram with fitted gaussian distribution for cortisone values
**Figure S4:** Log normal transformed frequency histogram with fitted gaussian distribution for cortisone values
**Figure S5:** Normal distribution probability plot for hydrocortisone/cortisone ratio to determine the parametric range
**Figure S6:** Box and Whisker Plot for hydrocortisone/cortisone ratio (Gender Comparison) between female (n = 447), gelding (n = 475), and male (n = 228) horses.
**Figure S7:** Box and Whisker Plot for hydrocortisone/cortisone ratio (Gender Comparison) between female (n = 228), gelding (n = 228) and male (n = 228) horses
**Figure S8:** Hydrocortisone concentration during the administration for TACA for Horse 3 (Blue line), 4 (Green Line) and 5 (Grey Line) (Time 0 = time of administration). Yellow dotted line represents the proposed lower hydrocortisone threshold
**Figure S9:** HC/C values (blue) following TACA administration (20 mg IA) for Horse 3 with comparison to IRLs (red) and PRLs (yellow).
**Figure S10:** HC/C values (blue) following TACA administration (20 mg IA) for Horse 5 with comparison to IRLs (red) and PRLs (yellow)
**Figure S11:** Amount of hydrocortisone removed from blank plasma using DCM:EtOH (90:10 v/v)

## Data Availability

The data that support the findings of this study are available from the corresponding author upon reasonable request.
