# Supplementary material for: Measurements of hydrocortisone and cortisone for longitudinal profiling of equine plasma by liquid chromatography–tandem mass spectrometry
Source: Drug Test Anal. 2022 Mar 4;14(5):943–52. doi: 10.1002/dta.3244 (PMC9545025; doi:10.1002/dta.3244)
Supplement: Supplementary file 1 — Table S1: Plasma spikes for calibration of Hydrocortisone (HC) Table S2: Plasma Spikes for Calibration of Cortisone (C) Table S3: Plasma Spikes for Calibration of Triamcinolone Acetonide (TACA) Table S4: Removal of endogenous compounds using DCM: EtOH (90:10) Table S5: Reference Population Data for HC: Table S6: Reference Population Data for C: Figure S1: Frequency Histogram with fitted gaussian distribution for Hydrocortisone values Figure S2: Log normal transformed frequency histogram with fitted gaussian distribution for hydrocortisone values Figure S3: Frequency histogram with fitted gaussian distribution for cortisone values Figure S4: Log normal transformed frequency histogram with fitted gaussian distribution for cortisone values Figure S5: Normal distribution probability plot for hydrocortisone/cortisone ratio to determine the parametric range Figure S6: Box and Whisker Plot for hydrocortisone/cortisone ratio (Gender Comparison) between female (n = 447), gelding (n = 475), and male (n = 228) horses. Figure S7: Box and Whisker Plot for hydrocortisone/cortisone ratio (Gender Comparison) between female (n = 228), gelding (n = 228) and male (n = 228) horses Figure S8: Hydrocortisone concentration during the administration for TACA for Horse 3 (Blue line), 4 (Green Line) and 5 (Grey Line) (Time 0 = time of administration). Yellow dotted line represents the proposed lower hydrocortisone threshold Figure S9: HC/C values (blue) following TACA administration (20 mg IA) for Horse 3 with comparison to IRLs (red) and PRLs (yellow). Figure S10: HC/C values (blue) following TACA administration (20 mg IA) for Horse 5 with comparison to IRLs (red) and PRLs (yellow) Figure S11: Amount of hydrocortisone removed from blank plasma using DCM:EtOH (90:10 v/v) [file DTA-14-943-s001.pdf]

Measurements of Hydrocortisone and Cortisone for Longitudinal Profiling of Equine Plasma by LC-MS/MS: Supporting Information

Kathy Tou[1], Adam Cawley [2], Chris Bowen[3], Kireesan Sornalingam[2], Shanlin Fu[1]

[1]Centre for Forensic Science, University of Technology Sydney, Broadway, NSW, Australia 2007
[2] Australian Racing Forensic Laboratory, Racing NSW, Sydney NSW, Australia 2000
[3] Shimadzu Scientific Instruments (Australasia), Sydney, NSW Australia 2116

SI Section 1: Method Validation Method for Hydrocortisone, Cortisone and TACA quantification

Linearity

SI Table S1: Plasma spikes for calibration of Hydrocortisone (HC)

Spike Concentration (ng/mL)	Analyte Solution Used	Amount (μL)
0	-	-
1	Working 100 ng/mL	20
2	Working 100 ng/mL	40
5	Working 100 ng/mL	100
10*	Working 100 ng/mL	200
50*	Working 500 ng/mL	200
100*	Working 500 ng/mL	400
150	Stock 2 μg/mL	150
200	Stock 2 μg/mL	200

*QC Samples

SI Table S2: Plasma Spikes for Calibration of Cortisone (C)

Spike Concentration (ng/mL)	Analyte Solution Used	Amount (μL)
0	-	-
1*	Working 100 ng/mL	20
2	Working 100 ng/mL	40
5*	Working 100 ng/mL	100
10*	Working 100 ng/mL	200

*QC Samples

SI Table S3: Plasma Spikes for Calibration of Triamcinolone Acetonide (TACA)

Spike Concentration (ng/mL)	Analyte Solution Used	Amount (µL)
0	-	-
0.05	Working 10 ng/mL	10
0.1*	Working 10 ng/mL	20
0.2	Working 10 ng/mL	40
0.5	Working 10 ng/mL	100
1*	Working 100 ng/mL	20
2*	Working 100 ng/mL	40
5	Working 100 ng/mL	100
10	Working 100 ng/mL	200

*QC Samples

The accuracy of the regression analysis was assessed by analysing the y-residuals from the linearity curve for each of the compounds. This was completed by analysing each replicate of the linearity curve analysing the residual with the expected concentration determined from the linearity and the calculated concentration determined by LabSolutions software. This method is used to account for the differences within the spiking method. To determine if the method is accurate, all points were expected to fall within a range of ± 1. If the residuals fell outside this range, there would be the presence of bias in the method.

Sensitivity:
Sensitivity was assessed for HC, C and TACA, using a visual comparison of the spikes at a lower concentration in comparison to the plasma calibrators. The LOD and LOQ were estimated by a visual comparison of the smallest peak (usually the tallest noise peak) of the lowest concentration that the instrument could detect for each compound. Using the signal-to-noise ratio (*S/N*), comparison could be made for a *S/N* of 3 for LOD and a *S/N* of 10 for LOQ. Concentrations of 0.05, 0.1, 0.2 and 0.5 ng/mL were chosen for HC and C whilst for TACA, concentrations of 0.01, 0.02, 0.03, 0.04, 0.05 and 0.1 ng/mL.

Accuracy:
Accuracy was estimated by the calculation of the precent relative error (%RE) from the mean concentration of each QC sample compared to the theoretical concentration. Each QC was completed in duplicate.

Precision:
Precision was estimated as %RSD using the average concentrations of duplicate QC samples. Precision was deemed to be acceptable within < 20%.

Recovery:
Recovery was assessed by comparing pre- and post-SPE equine plasma spike replicates (n=7) at the QC levels with recovery expected to be higher than 50%.

Matrix Effects:
Matrix effects were assessed by comparing post-SPE spiked plasma samples to neat standards (n=7). Matrix effects greater than 100%, indicated ion enhancement, while less than 100% indicated ion suppression.

Dilution Factor:
Dilution factor was assessed using concentrations of 7 replicated samples that represented either a 1 mL or 0.5 mL of solution. The chosen concentration of HC for the spike was 300 ng/mL as dilution for 1 mL (1 in 2) and 0.5 mL (1 in 4) sample volume would fall within the chosen calibration range. The concentration determined by the instrument was then multiplied by the relevant dilution factor to obtain the sample concentration. For the dilution to be deemed acceptable, this required a %RE of $\leq$ 15%.

Stability:
Stability was assessed over a 3-month period at two different temperatures: 4°C and -20°C in the DCM: EtOH surrogate matrix. Each QC sample was spiked in duplicate with the analyte considered stable if the concentration was within ± 20 ng/mL.

SI Section 2: Results and Discussion:

SI Table S4: Removal of endogenous compounds using DCM : EtOH (90:10)

Compound	Area Response	Area Remaining	Percentage Removed (%)
**Hydrocortisone**	53043146	4895	99.99
**Cortisone**	1795098	0	100

SI Table S5: Reference Population Data for HC:

**Hydrocortisone**	
**Mean (ng/mL)**	56
**Median (ng/mL)**	54
**Standard Deviation**	21
**Q1**	41
**Q2**	54
**Q3**	69
**IQR**	27

SI Table S6: Reference Population Data for C:

**Cortisone**	
**Mean (ng/mL)**	3.6
**Median (ng/mL)**	1.5

**Standard Deviation**	3.5
**Q1**	2.4
**Q2**	3.5
**Q3**	4.5
**IQR**	2.0

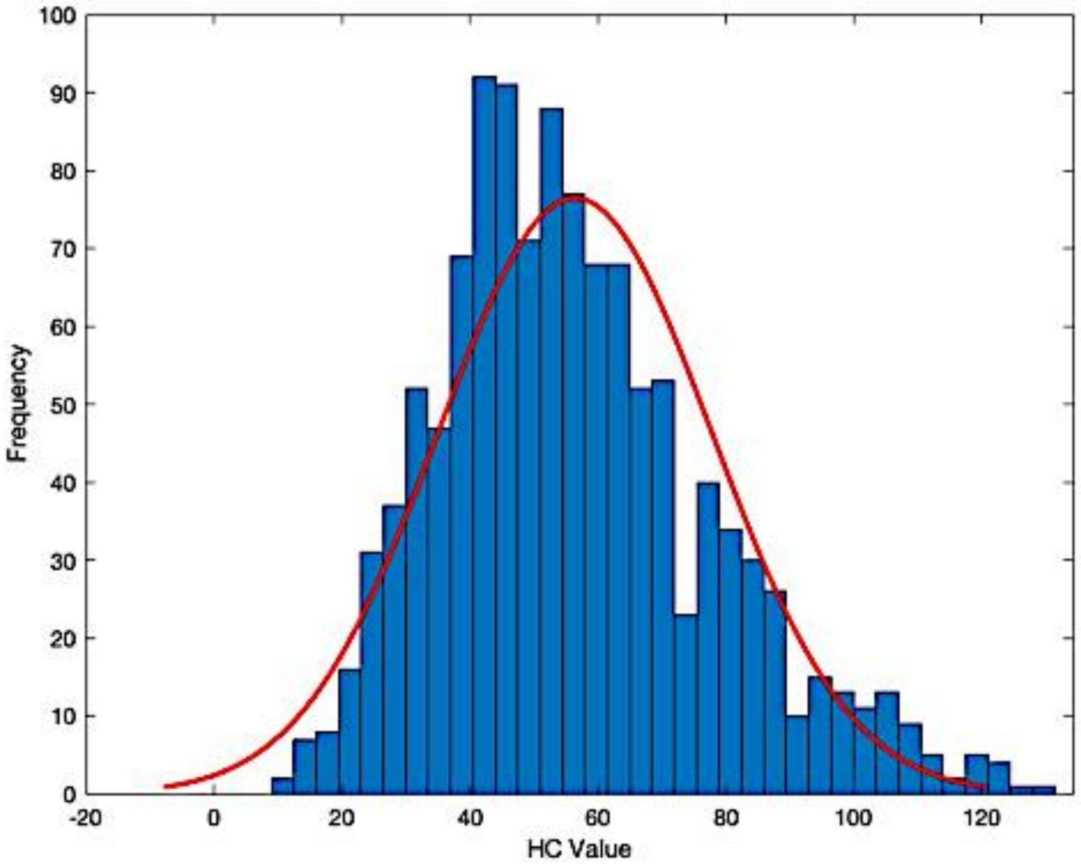

SI Figure S1: Frequency Histogram with fitted gaussian distribution for Hydrocortisone values

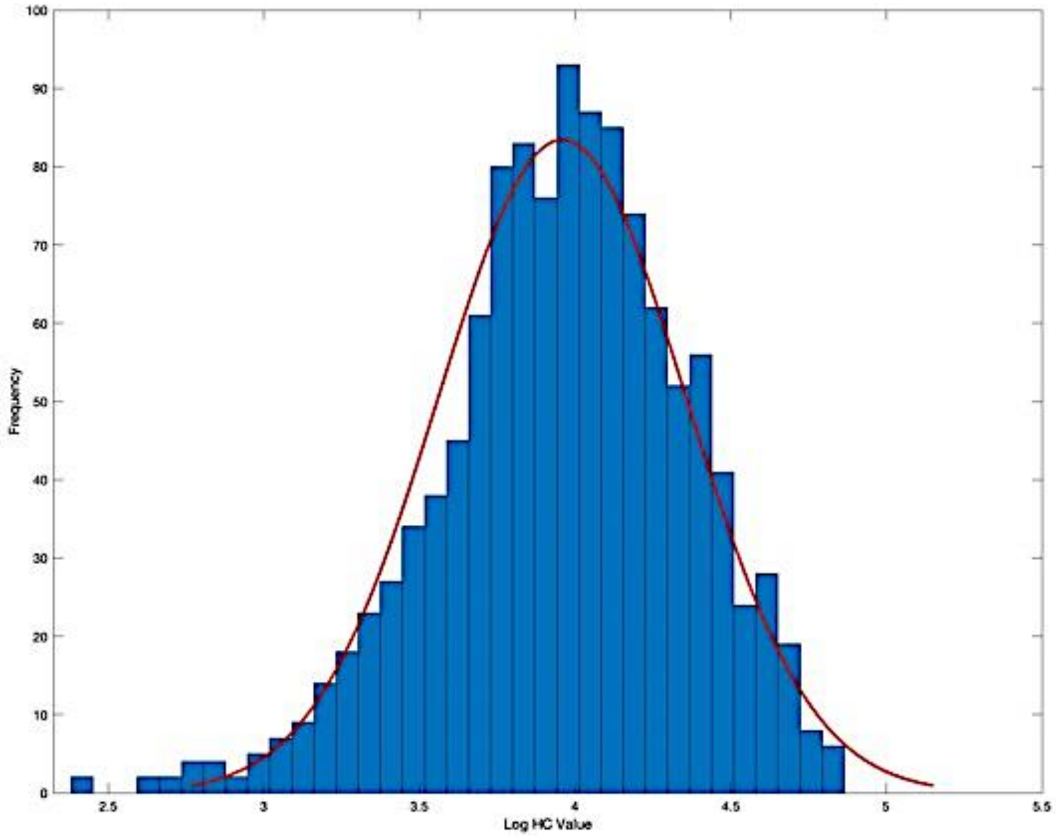

SI Figure S2: Log normal transformed frequency histogram with fitted gaussian distribution for hydrocortisone values

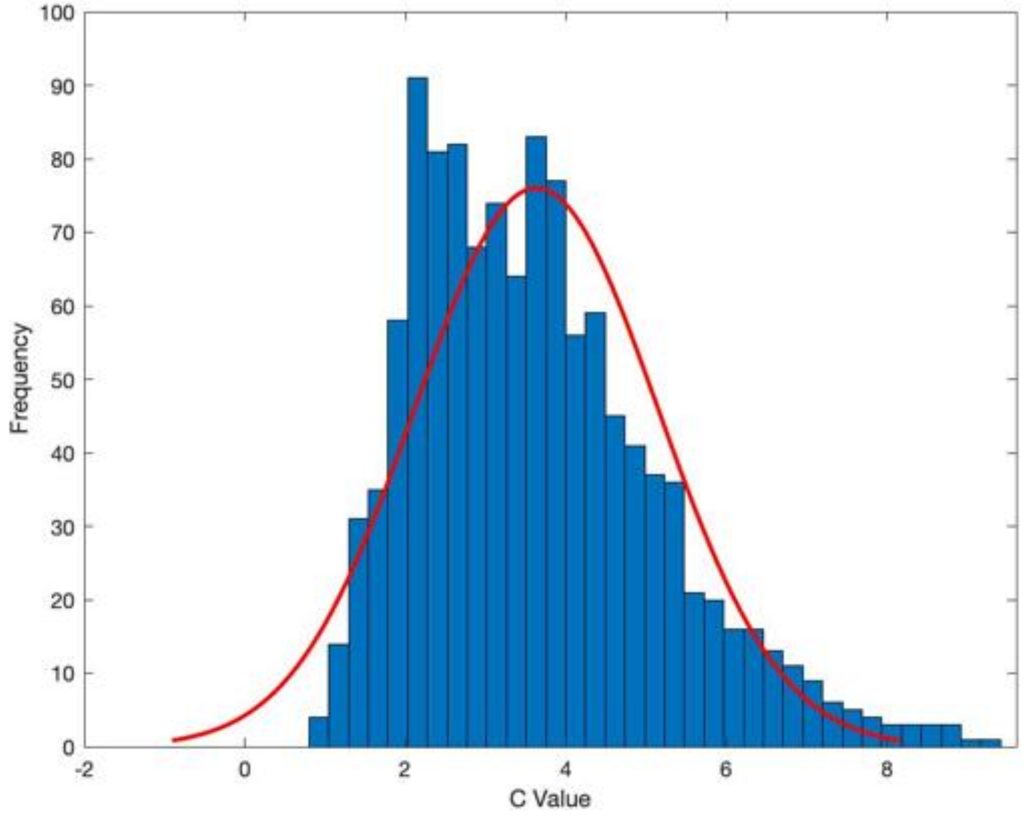

SI Figure S3: Frequency histogram with fitted gaussian distribution for cortisone values

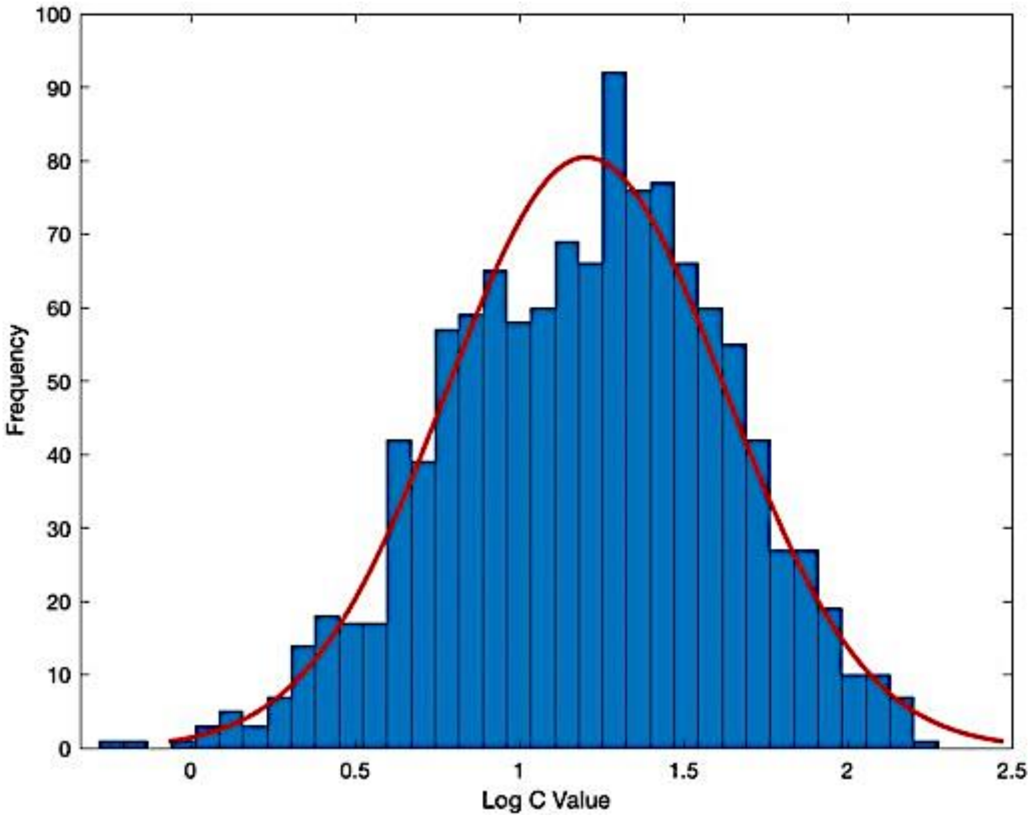

SI Figure S4: Log normal transformed frequency histogram with fitted gaussian distribution for cortisone values

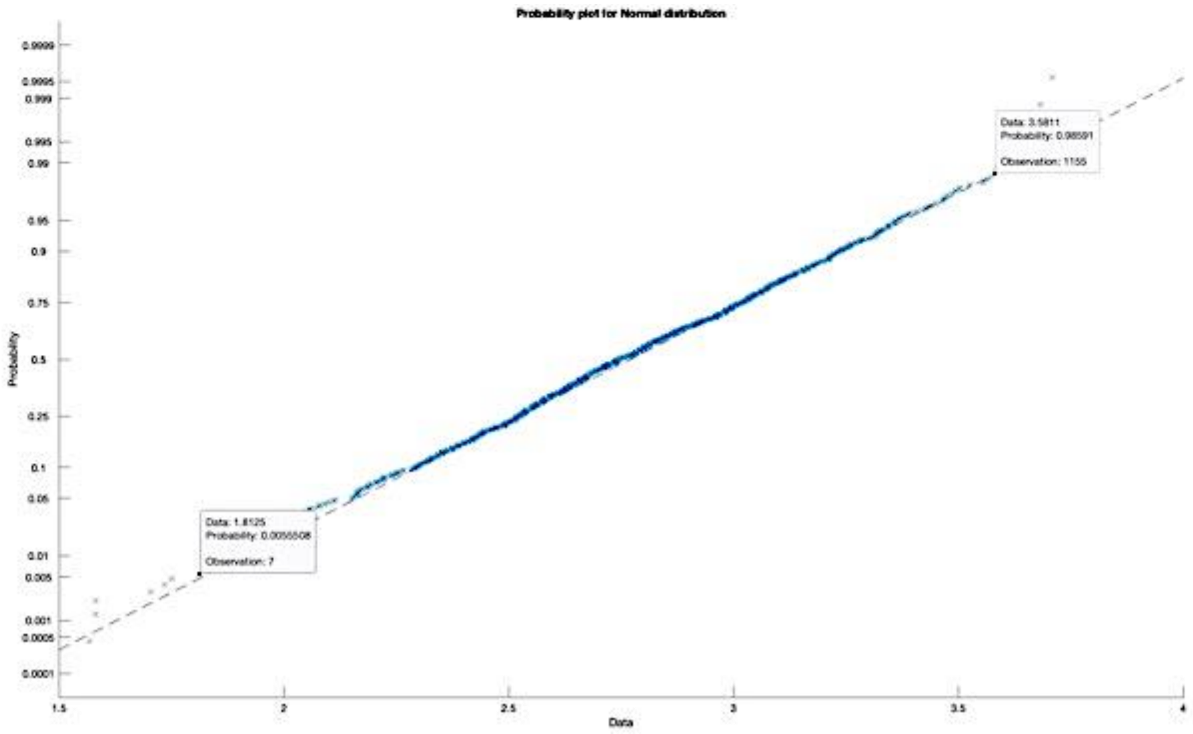

SI Figure S5: Normal distribution probability plot for hydrocortisone/cortisone ratio to determine the parametric range

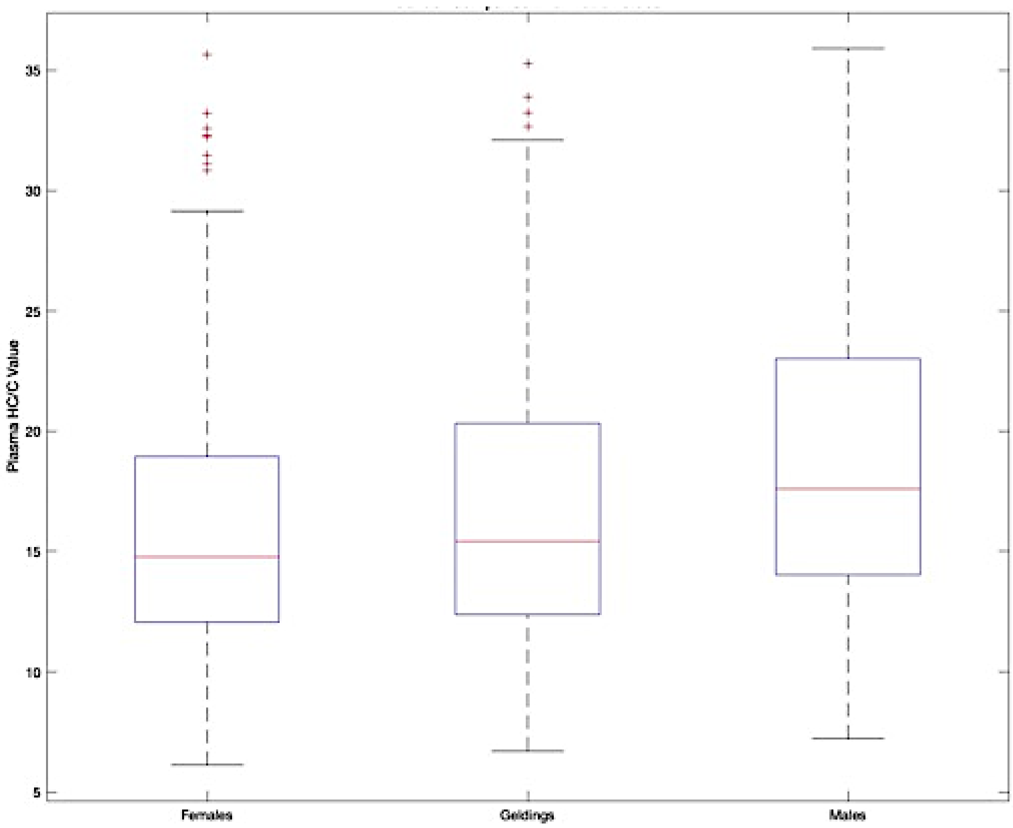

SI Figure S6: Box and Whisker Plot for hydrocortisone/cortisone ratio (Gender Comparison) between female (n=447), gelding (n=475), and male (n=228) horses.

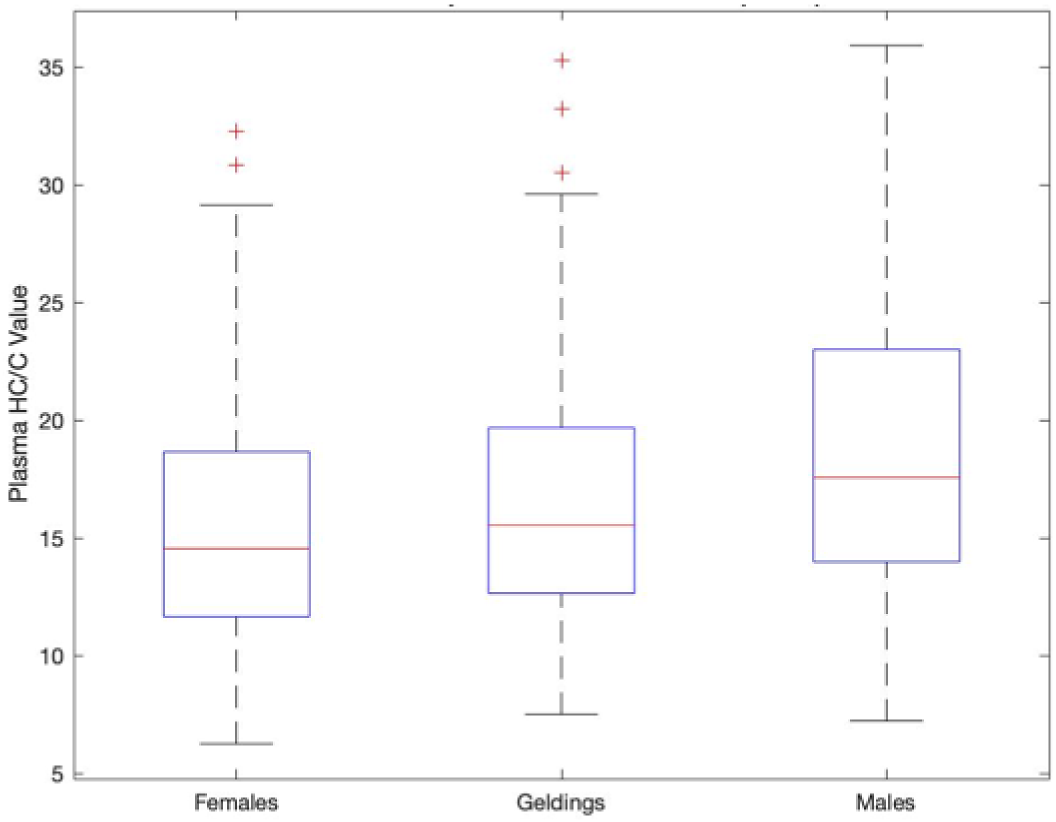

SI Figure S7: Box and Whisker Plot for hydrocortisone/cortisone ratio (Gender Comparison) between female (n=228), gelding (n=228) and male (n=228) horses

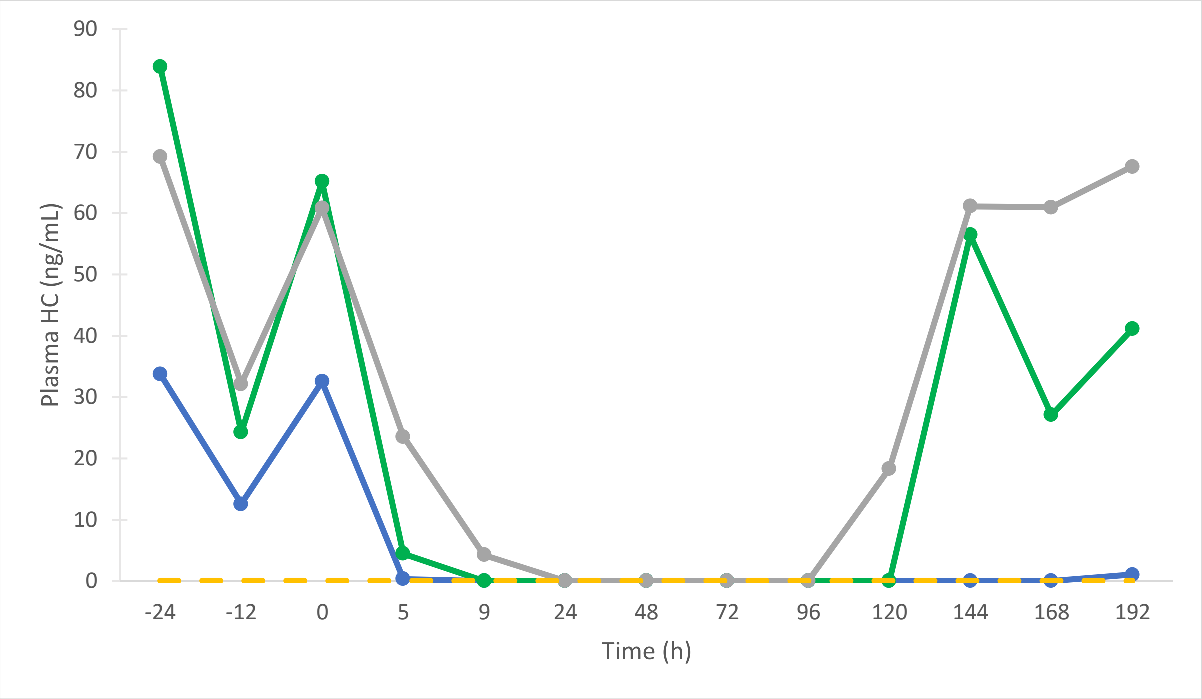

SI Figure S8: Hydrocortisone concentration during the administration for TACA for Horse 3 (Blue line), 4 (Green Line) and 5 (Grey Line) (Time 0 = time of administration). Yellow dotted line represents the proposed lower hydrocortisone threshold

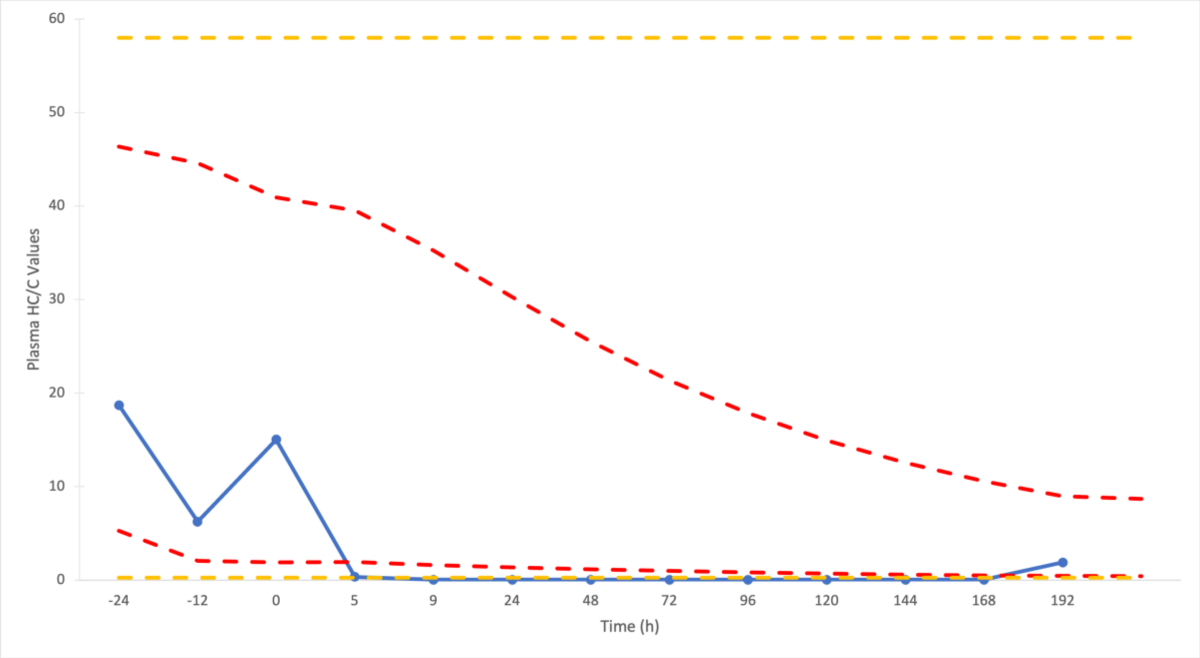

SI Figure S9: HC/C values (blue) following TACA administration (20 mg IA) for Horse 3 with comparison to IRLs (red) and PRLs (yellow).

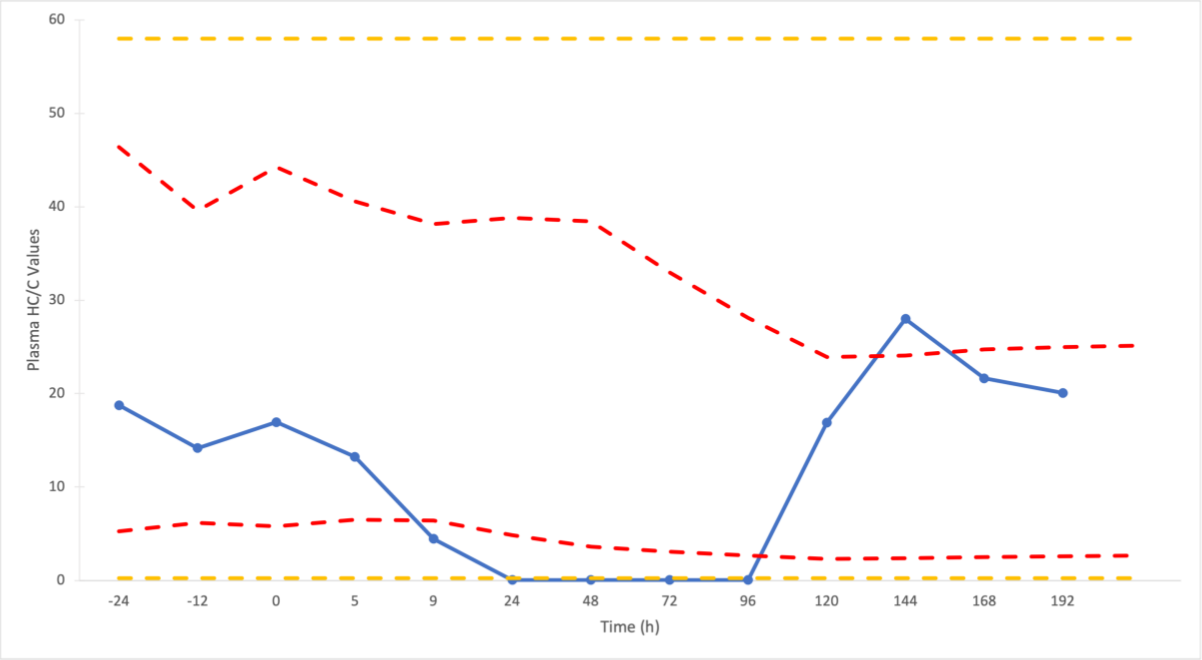

SI Figure S10: HC/C values (blue) following TACA administration (20 mg IA) for Horse 5 with comparison to IRLs (red) and PRLs (yellow)

SI Section 3: Surrogate Matrix Optimisation:

A surrogate matrix was used as the y-intercept for HC using standard addition for the calibration range was very high (greater than 6). This high y-intercept is not considered suitable as this study required an accurate method to determine low levels of HC as the TACA administration resulted in down-regulation of HC. So, whilst the calibration range using standard addition was acceptable, the high y-intercept wasn't therefore the need for a surrogate matrix for an accurate calibration was necessary.

Various methods were explored to optimise the surrogate matrix including solid phase extraction (SPE) and liquid-liquid extraction (LLE). Blank plasma from a pooled collection of plasma known to not contain any exogenous drugs was used to produce the surrogate matrix. For LLE, 3 mL of blank plasma was transferred from the pooled amount into screw top test tubes, 4 mL of di-isopropyl ether (DIPE) was added to each tube and rotated on the mixer for 20 minutes at medium speed allowing for complete inversion between the plasma and the DIPE. Each tube was further centrifuged for 3000 rpm for 10 minutes then the aqueous plasma layer from each tube was extracted into a clear plastic bottle and stored at 4 °C until use. This process was completed a total of 3 times for each bottle of stripped plasma that underwent LLE using DIPE.

Another 2 sets of LLEs were completed using either dichloromethane (DCM) and methanol (MeOH) in a 90:10 ratio or dichloromethane (DCM) and ethanol (EtOH) in a 90:10 ratio. For each of these mixtures, 3 mL of blank plasma was transferred from the pooled amount into screw top test tubes. Into each test tube, 3 mL of either DCM:MeOH or DCM:EtOH was

added, rotated on a mixer for 20 minutes at medium speed to allow for complete inversion between the plasma and the extraction solution. Each tube was centrifuged at 3000 rpm for 10 minutes then the aqueous plasma layer from each tube was extracted into a clear plastic bottle and then stored at 4 °C until use.

For SPE, a clean-up carbon extraction cartridge from UCT (Bristol, PA, United States of America) was used. Cartridges were pre-conditioned with approximately 3 mL of purified water, 3 mL of blank plasma from the pooled amount was then transferred into each carbon cartridge. Plasma was allowed to run through the carbon cartridge using gravity to filter the plasma through the cartridge into a test tube.

Using HC as an example, LLE using three lots of DIPE removed 12%, SPE carbon cartridges removed 30% whilst LLE DCM:MeOH in a 90:10 ratio removed 90% of endogenous HC. The best method to produce the surrogate matrix utilised DCM:EtOH in a 90:10 ratio, which successfully removed 99% of the endogenous HC (SI Figure S11). The use of DCM:EtOH in a 90:10 ratio also allowed for the y-intercept to be reduced from originally 6.99 to 0.15 therefore allowing for a more accurate calibration range to detect lower levels of HC. This result was consistent with previous studies conducted by Popot *et al.* for equine urine which is advantageous as this method was used to establish the hydrocortisone threshold in urine.

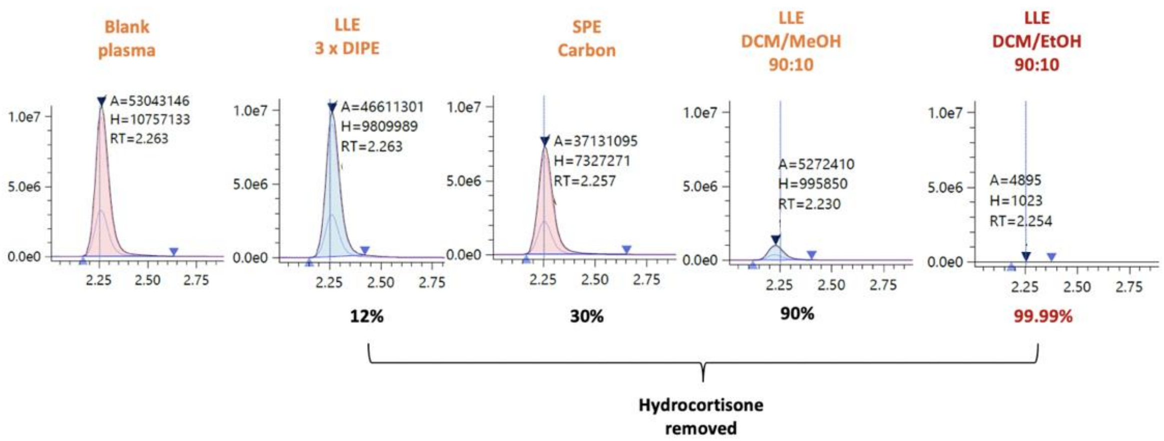

SI Figure S11: Amount of hydrocortisone removed from blank plasma using DCM:EtOH (90:10 v/v)
